# Sexual History Documentation and Screening in Adolescent Females with Suicidal Ideation in the Emergency Department

**DOI:** 10.3390/ijerph192013018

**Published:** 2022-10-11

**Authors:** Tatyana Vayngortin, Katie Clark, Kathryn Hollenbach

**Affiliations:** 1 Division of Emergency Medicine, Rady Children’s Hospital San Diego, San Diego, CA 92123, USA; 2 Department of Pediatrics, San Diego School of Medicine, University of California, La Jolla, CA 92093, USA

**Keywords:** adolescent, pediatric emergency department, depression, suicidal ideation, reproductive health

## Abstract

Adolescents with mental illness often seek care in the emergency department (ED) and are more likely to engage in risky behaviors such as substance abuse and unprotected sex, increasing their risk of sexually transmitted infections (STI), unintended pregnancy, and non-consensual sex. This was a retrospective study of 312 females, aged 13–17 years, presenting to the pediatric ED with the chief complaint of suicidal ideation from February to May 2018. Electronic medical records were reviewed for demographics, psychiatric history, sexual history, and testing for pregnancy or STI. The primary outcome was the documentation of the presence or absence of prior sexual activity. Secondary outcomes included documented aspects of sexual history and pregnancy or STI testing performed in the ED. Of the 312 eligible patients, 144 (46.2%) had a documented sexual history, and of those 50 (34.7%) reported being sexually active. Sexual history documentation was not associated with patient age, race, ethnicity, insurance, or the gender of the ED provider. A history of anxiety and a recent suicide attempt were associated with a lack of sexual history documentation (*p* = 0.03). Of the sexually active patients, 28 (56%) had documentation of contraception use. Pregnancy testing was performed in 67.3% of all patients and 80% of sexually active patients. Only 10 patients had STI testing in the ED, with most testing occurring in those with sexual history documentation (*p* = 0.007). In conclusion, more than half of females with suicidal ideation in our ED had no documentation of sexual history, and when documentation was completed, it was often missing important elements, including screening for pregnancy, STI, non-consensual sex, and contraception use. Since the ED visit provides an important opportunity to address the reproductive health needs of this high-risk population, further work is needed to determine ways to improve provider documentation and sexual health screening.

## 1. Introduction

Adolescence is a formative time and can be highly stressful due to the numerous physical, emotional, and psychosocial changes that occur. This time is often accompanied by self-discovery, sexual exploration, and at times, the onset of mental illnesses. The vast majority of mental illnesses begin before the end of adolescence, though many disorders remain undiagnosed and without treatment for several years [1]. The most common mental illnesses in teens are anxiety, mood, attention, and behavior disorders [2]. The World Health Organization estimates that roughly 15% of all adolescents experience a mental illness, and studies suggest that about 2/3 remain untreated [1]. Suicide is the second leading cause of death among young people aged 15–24 years, and the prevalence of mental illness and suicide attempts have increased during the SARS-CoV-2 pandemic [3].

Anxiety and mood disorders are two to three times more prevalent in female adolescents than in male adolescents [2]. Adolescents with mental illness often engage in high-risk behavior, including risky sexual practices and substance abuse, which increases their risk of unintended pregnancy and sexually transmitted infections (STI) [4]. Some are also at increased risk of STI due to sequelae of sexual abuse [4]. Studies have found that in adolescents, depression symptoms in particular are associated with risky sexual activity, such as unprotected sex [5,6]. Moreover, depressed mood and lack of motivation may be a barrier to seeking healthcare and behavioral change.

Adolescents who report using the ED as their usual source of care are less likely to have primary care visits and may miss opportunities for anticipatory guidance and reproductive health screening [7]. In a national survey of adolescents, Wilson et al. found that those with lower socioeconomic status, lack of insurance, mental health problems, and substance abuse were more likely to seek care in the ED [7]. Therefore, reproductive health screening and counseling are especially important in this vulnerable population. Depressed adolescent females often present to the ED due to suicidal ideation, and there are limited data on sexual health screening in this growing patient population. This study examined sexual history documentation and the frequency of pregnancy and STI testing in adolescent females presenting to the ED with suicidal ideation.

## 2. Materials and Methods

This was a retrospective chart review of 312 females, aged 13–17, presenting to the pediatric ED with a chief complaint of suicidal ideation from February to May 2018. The study occurred at Rady Children’s Hospital San Diego, which serves as the region’s primary and tertiary children’s hospital. It is an academic medical center affiliated with the University of California, San Diego. The 63-bed free-standing pediatric ED has about 100,000 annual visits, approximately 15% of which are adolescents. The majority of patients are Hispanic and have government insurance. The ED is staffed by pediatric emergency medicine (PEM) physicians, PEM fellows, and general pediatricians. Trainees include residents from pediatrics, emergency medicine, family medicine, and medical students. 

Patients were excluded if they were emergency severity index triage level 1 or had documented altered mental status or developmental or cognitive delay, as they would not be able to provide a sexual history. Electronic medical records, including physician and social work notes, were reviewed for demographics (age, sex, race, ethnicity, and insurance), psychiatric diagnoses, sexual history documentation, and pregnancy and STI testing. The primary outcome was sexual history documentation, defined as the documentation of the presence or absence of prior sexual activity. Secondary outcomes included aspects of the documented sexual history, including contraception use, the last sexual encounter, and a history of STI or pregnancy, non-consensual sex, and trafficking. We also examined whether pregnancy and STI testing were performed during the ED encounter.

Demographic and other characteristics of the study population were compared according to sexual history documentation status, using the chi square or Fisher’s exact tests for categorical variables and t-tests for continuous variables (STATA 16.0, StataCorp LLC, College Station, TX, USA). This study was approved by the University of California San Diego Human Research Protection Program and the Rady Children’s Hospital–San Diego (RCHSD) Research Office. 

## 3. Results

Of the 320 eligible females seen in the ED during the study period, 8 were excluded for altered mental status, and 312 charts were reviewed. Table 1 displays the demographic characteristics of our eligible study population. The average age was 15.1 +/− 1.5 years, and the majority of patients were White or Hispanic. 

Less than half (*n* = 144, 46.2%) had a documented sexual history, and of those, 50 (34.7%) were sexually active (Table 2). Sexual history documentation was not associated with patient age, race, ethnicity, insurance, or the gender of the ED provider. A history of anxiety and a recent suicide attempt were associated with an absence of sexual history documentation (*p* = 0.03). Of the sexually active patients, 28 (56%) were asked about contraception use, and of those 25 (89.3%) reported using a method, most often condoms, followed by oral contraceptive pills (OCP). Only 6 of the 25 (24%) reported using a long-acting reversible contraceptive (LARC) method, including an intrauterine device or implant. 

Approximately three fourths (*n* = 239, 73.7%) of patients were asked about their history of abuse, including physical and sexual, usually by the social worker rather than the physician. Of those, 28% (*n* = 67) reported a history of abuse, with 39 reporting physical abuse and 28 reporting sexual abuse in childhood. A total of 17 patients reported recent rape, usually by a peer or family member. There were four patients that were asked about their history of trafficking by the social worker, with two of those reporting being trafficked. 

Of the sexually active patients, 20% (*n* = 10) were asked when their last sexual encounter occurred, and only 8% (*n* = 4) were asked about prior pregnancy. In total, 16% (*n* = 8) of sexually active patients were asked about their history of STI, with one patient reporting a prior STI. Upon review of prior laboratory results, four (1.3%) patients had prior positive chlamydia or gonorrhea tests. No one had prior positive syphilis or HIV tests. Pregnancy testing was performed in 67.3% of all patients and 80% of sexually active patients. Only 10 patients had STI testing in the ED, with most testing occurring in those with documented sexual activity (*p* = 0.007). 

## 4. Discussion

More than half of adolescent females presenting to our ED for suicidal ideation had no documentation of sexual history. For those that were asked about sexual history, documentation was often missing important elements such as contraception use, the most recent sexual encounter, non-consensual sex, and prior history of pregnancy or STI. Multiple studies have demonstrated that adolescents with mental illness are more likely to engage in risky sexual activity and have higher risk of STI and unintended pregnancy compared to those without mental illness [8,9,10]. Therefore, obtaining a sexual history in this population is important for assessing reproductive health risks.

Ekstrom et al. also examined sexual history documentation in adolescents with mental health crises in a pediatric ED and found sexual history documentation in only 27% of their patients, which was less than in our population [11]. About one third (38%) of their sexually active patients had STI testing, compared to 20% of ours. The Centers for Disease Control and Prevention, the United States Preventive Services Task Force, and the American Academy of Pediatrics recommend screening all sexually active adolescents for STI at least annually [12]. Since adolescents with mental illness often have less access to primary care and have higher rates of ED utilization, STI screening should be considered at their ED visit. Moreover, urine chlamydia/gonorrhea testing can easily be collected with the urine pregnancy test. Studies have found that adolescents consider the ED an acceptable site for STI screening [13]. 

About half of sexually active patients were asked about contraception use, and most reporting using condoms or OCPs. While condoms are effective at preventing STI, they have a high failure rate (18%) in preventing pregnancy based on typical use [14]. Winner et al. found that adolescents are twice as likely to have an unintended pregnancy when using short-acting methods such as OCPs, patches, or rings compared to adults [15]. As urine pregnancy testing is easy to obtain and cost-effective, it should be considered in all adolescent females presenting to the ED with behavioral health complaints. Adolescent females should be asked about contraception use and counseled on contraceptive methods. Moreover, they should be asked about the date of their last sexual encounter to assess whether they would be candidates for emergency contraception if they desire to prevent pregnancy, as these medications are only effective if used within 5 days.

We found that sexual history documentation was not associated with patient demographic factors. This is in contrast to other studies that found higher rates of sexual history documentation and STI screening in non-Hispanic Black patients, older adolescents, and those with public insurance [16,17]. Our results may have differed due to our patient population being predominantly White and Hispanic, with less African-American patients than reported in other studies. Interestingly, we found that a diagnosis of anxiety was associated with lack of sexual history documentation. This may be due to providers seeking to avoid causing additional distress to anxious patients by asking them sexual history questions. Patients reporting recent suicide attempts were also less likely to be asked about their sexual history. These patients may have less history-taking in general, as providers already know their disposition will be inpatient psychiatric admission, leading to a more rapid and brief evaluation.

We reviewed social work notes in addition to physician notes, as these are usually more detailed and contain more psychosocial history than physician notes. We found documentation about a history or absence of abuse in 73.7% of charts, almost always in social work notes, with about one fourth (28%) of patients reporting abuse. Child abuse is an adverse childhood experience that is associated with worse mental and physical health outcomes [18]. Therefore, all patients with mental illness should be screened for a history of abuse. There were 17 patients that reported recent rape. This highlights the importance of asking teens about non-consensual sex to assess the need for medical evaluation as well as for reporting to child welfare services and law enforcement. Providers should be familiar with the laws in their state regarding reporting, confidentiality, and statutory rape. Sexual violence is under-reported yet has tremendous consequences, including increased rates of mental illness, physical illness (chronic pelvic or abdominal pain and dysmenorrhea, among others), unintended pregnancy, STI, and substance abuse [19]. Patients reporting a recent sexual assault should undergo pregnancy and STI testing and should be offered emergency contraception and an empiric treatment for STIs [19]. Only a few (1.3%) of our patients were asked about trafficking. Trafficking is an important and under-recognized public health problem, with an estimated prevalence of 4–11% in high-risk groups in the United States [20]. Hurst et al. implemented a screening tool for sex trafficking in a pediatric ED and found a prevalence of 12% in high-risk patients. They identified that a history of child abuse and a prior suicide attempt increased the odds of trafficking [21]. We are likely missing an ideal opportunity to identify victims of trafficking in the pediatric ED, and screening must be improved.

There were several limitations to our study. We had a relatively small sample size (312 patients), and most patients were White or Hispanic, though this is representative of our patient population. The study was conducted at a single center, so the results may not be applicable to the general population. We only included females to explore the risk of unintended pregnancy, and we only included those presenting with suicidal ideation to focus on this high-risk group. Expanding to additional chief behavioral health complaints and the inclusion of males would likely identify additional high-risk behaviors and patients in need of reproductive healthcare. Given that we obtained a sexual history in less than half of our eligible target population, it is likely that the results here are underestimating the true scope of these issues. 

## 5. Conclusions

More than half of adolescent females presenting to our ED with suicidal ideation had no documentation of sexual history, and when documentation was present it was often incomplete. Since adolescents with mental illness often seek care in the ED, this provides an opportunity to address the reproductive health needs of this high-risk population. Further research is needed to determine methods to improve ED provider sexual history taking, documentation, and counseling as well as to increase pregnancy and STI testing.

## Figures and Tables

**Table 1:** Demographics, N = 312.

	Mean +/− SD
Age (years)	15.1 +/− 1.5
	*n* (%)
Race	
Hispanic	123 (39.4)
White	132 (42.3)
Black	23 (7.3)
Asian/Pacific Islander	26 (8.3)
Other	131 (42)
Insurance	
Private	146 (46.8)
Public	142 (45.5)
Uninsured	24 (7.7)

**Table 2:** Comparison of patients with and without sexual history documentation.

	Documented Sexual History (*n* = 144)	No Documented Sexual History (*n* = 168)	
	Mean (SD)	Mean (SD)	*p* value
Age (years)	15.2 (1.5)	14.9 (1.4)	0.08
	*n* (%)	*n* (%)	
Race			0.75
White	58 (40.3)	74 (44.1)	
Hispanic	55 (40.7)	68 (41.7)	
Black	13 (9)	10 (6)	
Asian/Pacific Islander	15 (10.4)	11(6.5)	
Other	58 (40.3)	73 (43.5)	
Insurance			0.70
Private	66 (45.8)	77 (45.8)	
Public	65 (45.1)	80 (47.6)	
Uninsured	13 (9)	11 (6.6)	
Recent suicide attempt	23 (16)	51 (30.4)	0.003
Anxiety diagnosis	56 (33.3)	65 (45.1)	0.033
Depression diagnosis	117 (81.3)	146 (86.9)	0.21
Female provider	76 (52.8)	95 (56.9)	0.47
Pregnancy test completed	100 (69.4)	110 (65.5)	0.46
STI testing completed	9 (6.3)	1 (0.6)	0.005

## Data Availability

Not applicable.
